# Supplementary material for: CRISPR/Cas9 targeted genetic screening in Physcomitrella identifies novel cell division genes
Source: Front Plant Sci. 2025 Dec 3;16:1653765. doi: 10.3389/fpls.2025.1653765 (PMC12708537; doi:10.3389/fpls.2025.1653765)
Supplement: Supplementary Figure 1 — Frameshift verification and initial phenotype observation in cyr1cyr2#1 and #2 frameshift lines. (A) Gene models for cyr1 and cyr2 genes, and frameshift mutations confirmed by sequencing in cyr1cyr2 #1 and cyr1cyr2 #2 moss lines. The gRNA target site is indicated with a blue box. (B) Membrane staining with 10 µM of lipophilic dye FM4-64. Nucleus and membranes in same color. Cyan arrowheads point to nuclei, red arrowheads point to cell walls. Note binucleated cell (2nd panel), multiple cell walls (3rd panel), and incomplete cell wall (4th panel) in the cyr1cyr2 #2 mutant. Scale, 10 µm. (C) Membrane staining in the adapted cyr1cyr2 #1 and cyr1cyr2 #2 lines after repeated culture. Cyan arrowheads point to multinuclei, red arrowheads point to defective cell walls, yellow arrowheads point to abnormal branching. Scale, 50 µm. (D) Number of cells with different phenotypes observed in the adapted cyr1cyr2 #1 and cyr1cyr2 #2 lines. [file DataSheet1.docx]

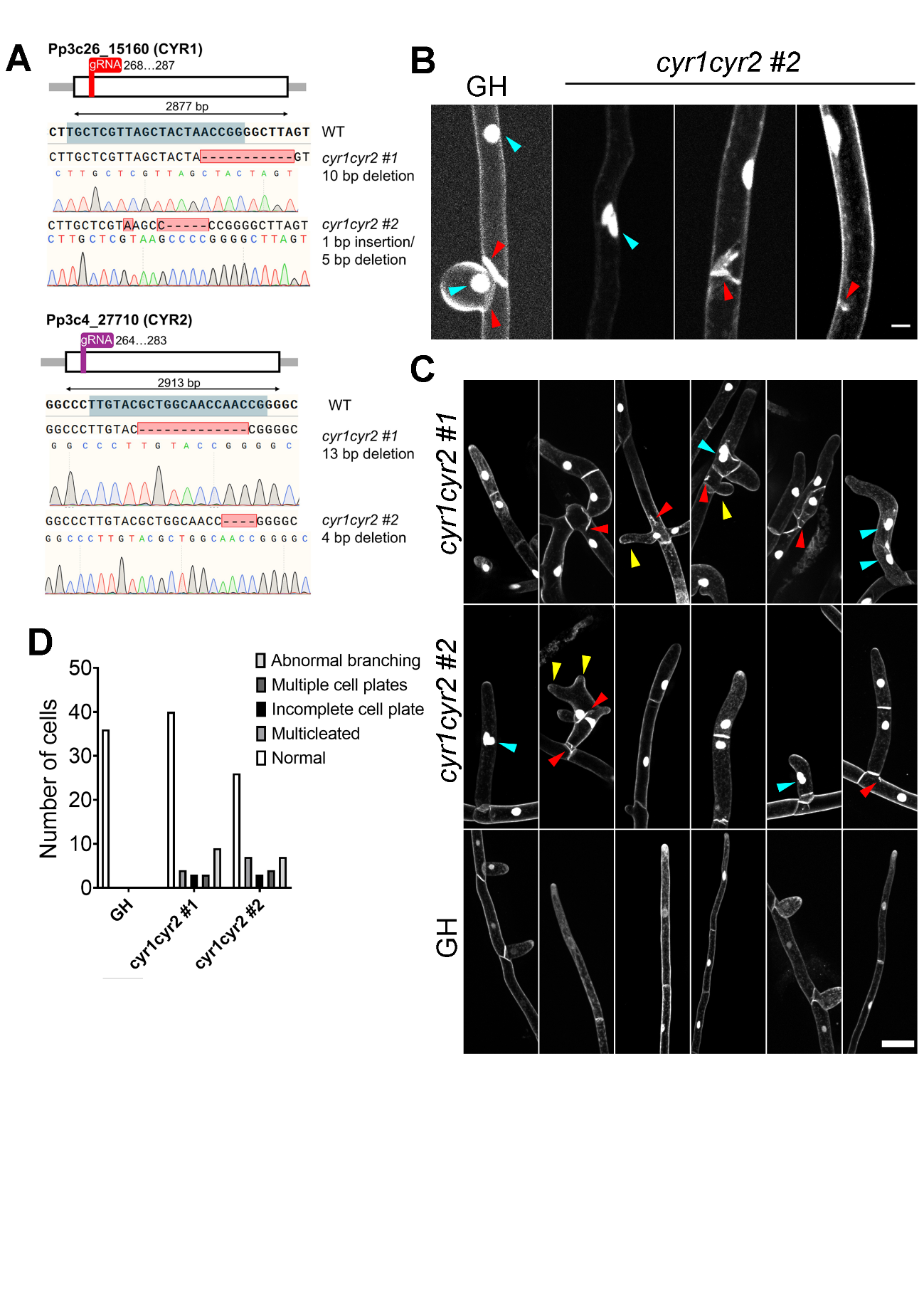


**Supplemental Figure 1. Frameshift verification and initial phenotype observation in *cyr1cyr2#1* and *#2* frameshift lines.** (**A**) Gene models for cyr1 and cyr2 genes, and frameshift mutations confirmed by sequencing in *cyr1cyr2 #1* and *cyr1cyr2 #2* moss lines. The gRNA target site is indicated with a blue box. (**B**) Membrane staining with 10 µM of lipophilic dye FM4-64. Nucleus and membranes in same color. Cyan arrowheads point to nuclei, red arrowheads point to cell walls. Note binucleated cell (2^nd^ panel), multiple cell walls (3^rd^ panel), and incomplete cell wall (4^th^ panel) in the *cyr1cyr2 #2* mutant. Scale, 10 µm. **(C)** Membrane staining in the adapted *cyr1cyr2 #1* and *cyr1cyr2 #2* lines after repeated culture. Cyan arrowheads point to multinuclei, red arrowheads point to defective cell walls, yellow arrowheads point to abnormal branching. Scale, 50 µm. **(D)** Number of cells with different phenotypes observed in the adapted *cyr1cyr2 #1* and *cyr1cyr2 #2* lines.


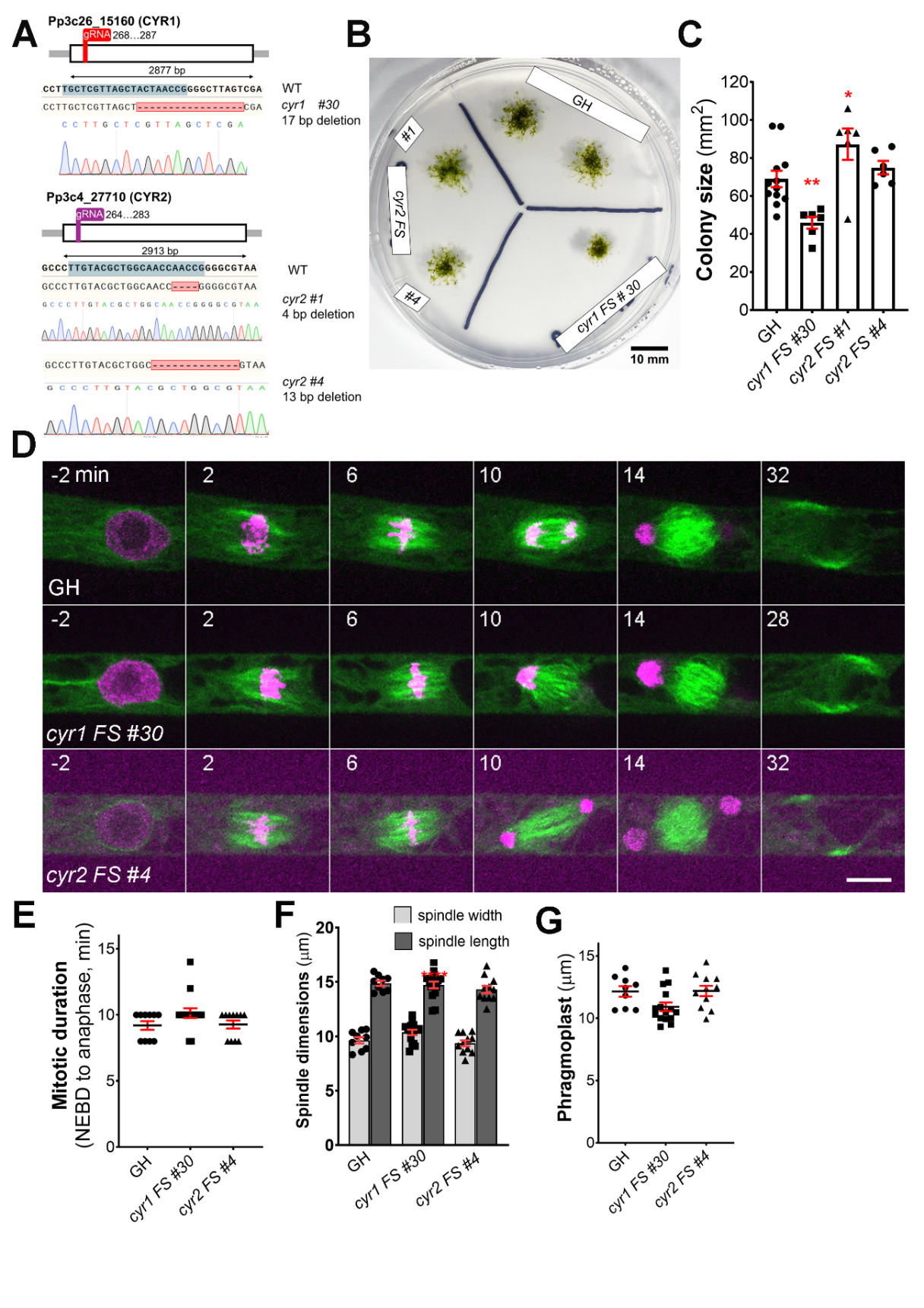
**Supplemental Figure 2. Frameshift verification and phenotype observation in *cyr1* and *cyr2* single frameshift lines.** (**A**) Gene models for cyr1 and cyr2 genes, and frameshift mutations confirmed by sequencing in *cyr1#30* and *cyr2 #1* and *#4* lines. The gRNA target site is indicated with a blue box. (B) Representative images of moss colonies after3 weeks of culture. Scale, 10 mm. (C) Colony size measured after 3 weeks of culture. Each data point corresponds to a single colony (mean ± SEM, **p=0.0089, *p=0.04 by one-way ANOVA with Dunnett’s multiple comparison test against GH) (**D**) Representative images of cell division in GH, *cyr1 FS #30*, and *cyr2 FS #4*. Time zero is set at NEBD, and single focal plane images are shown. Scale, 10 µm. (**E**) Mitotic duration calculated from NEBD to anaphase onset. Each data point corresponds to a single cell  (**F**) Spindle dimensions: spindle width (light gray bars) and spindle length (dark gray bars). Each data point corresponds to a single cell  (**G**) Phragmoplast length in GH, *cyr1 FS #30*, and *cyr2 FS #4*. Each data point corresponds to a single cell. No statistically significant difference was found in *cyr1 FS #30*, and *cyr2 FS #4* (G-F) against control (GH)


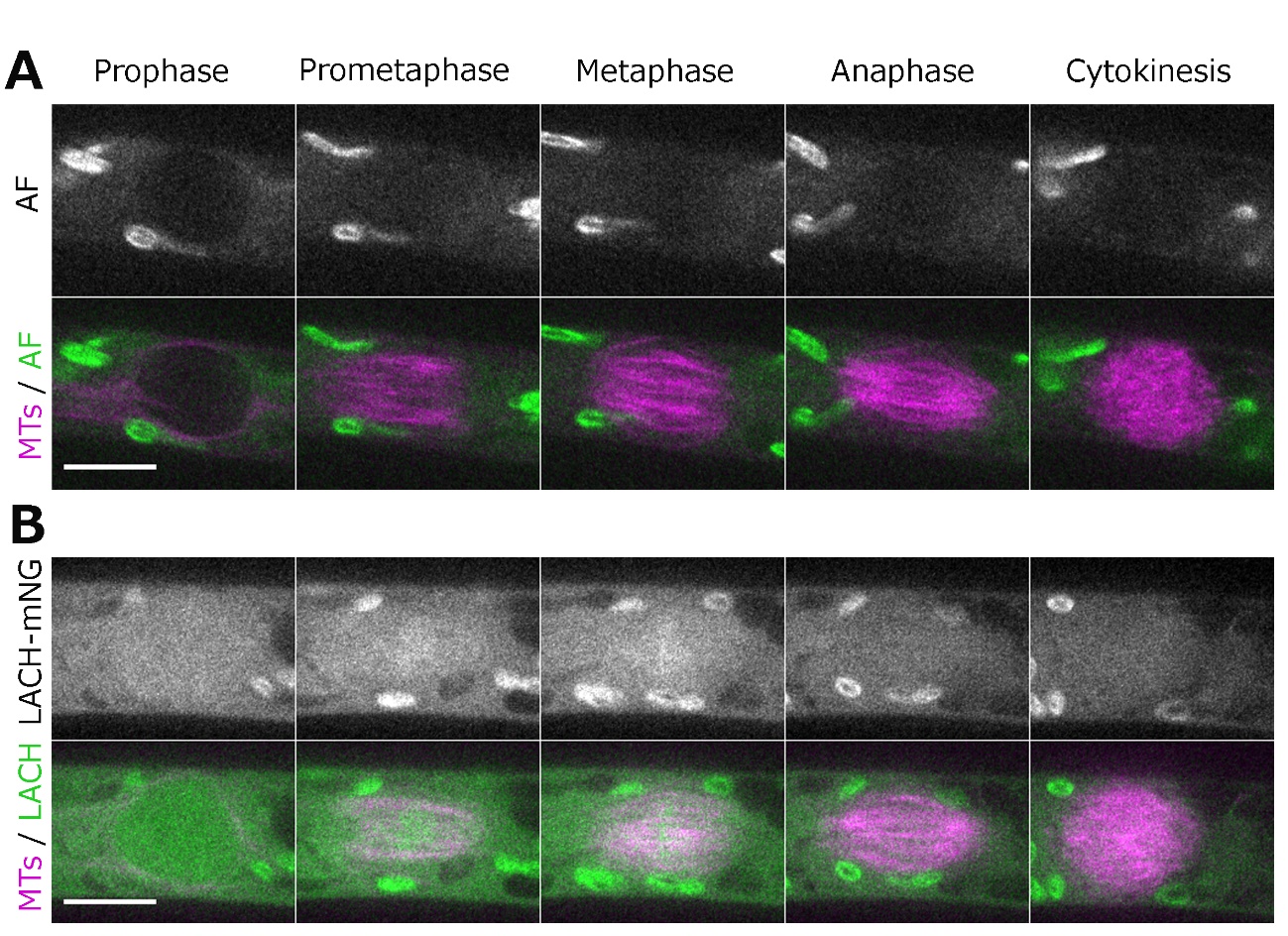


**Supplemental Figure 3. Localization of LACH-mNeonGreen during mitosis.** (**A**) Live imaging of mitosis in caulonemal apical cells, expressing mCherry-tubulin as a microtubule marker (magenta). AF panel shows cell autofluorescence background, including chloroplast autofluorescence. Scale, 10 µm. (**B**) Live imaging of *P. patens* protonemal apical cells expressing mCherry-tubulin (magenta) and LACH-mNeonGreen (green). Scale, 10 µm.


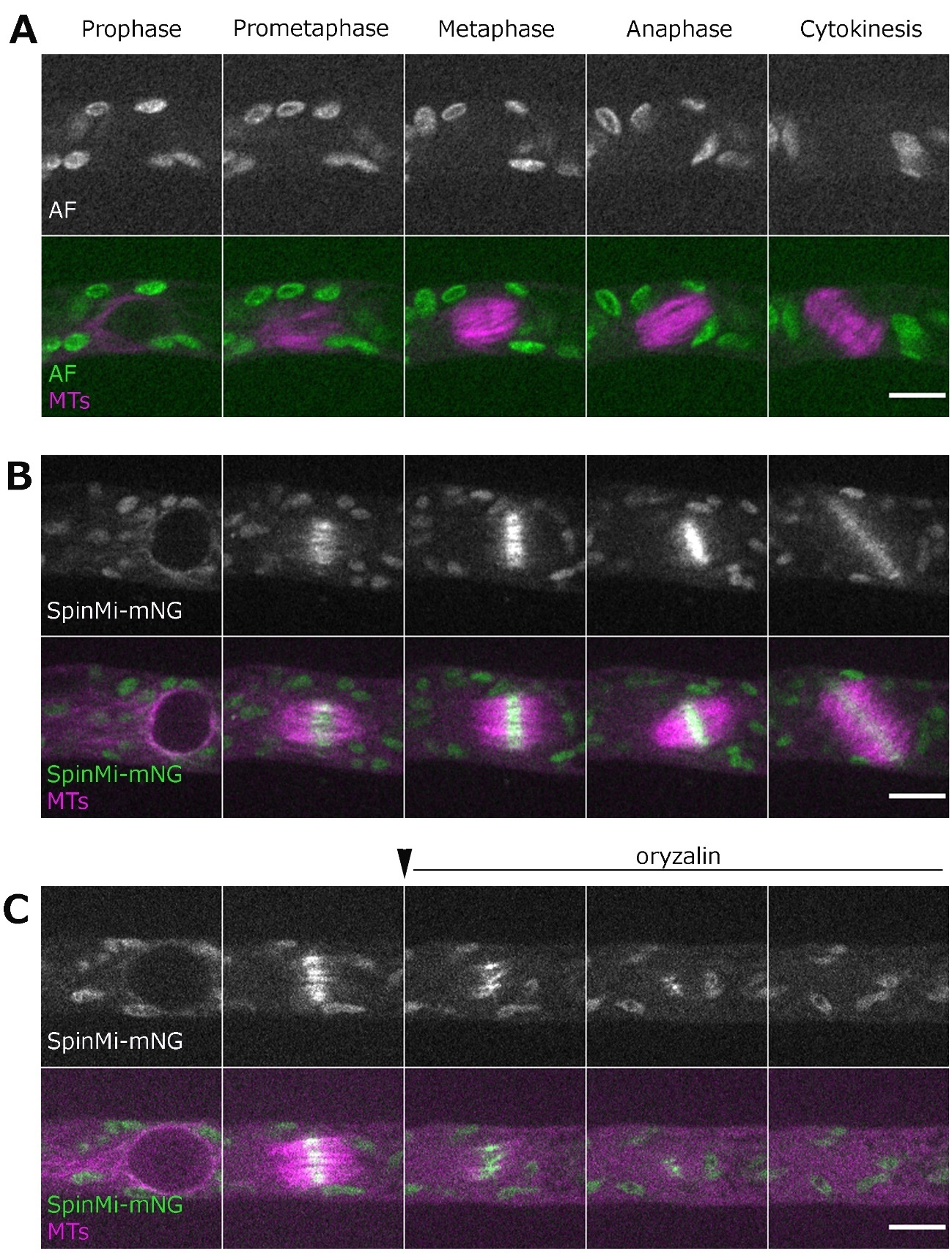


**Supplemental Figure 4. Localization of SpinMi during cell division.** Live imaging of *P. patens* caulonemal apical cells expressing (**A**) mCherry-tubulin and (**B**) mCherry-tubulin and SpinMi-mNeonGreen. AF panel shows cell autofluorescence background, including chloroplast autofluorescence. (**C**) Microtubule-depolymerizing drug oryzalin, at final concentration 20 µM, was added during prometaphase to the cell expressing mCherry-tubulin/SpinMi-mNeonGreen. Scale, 10 µm.


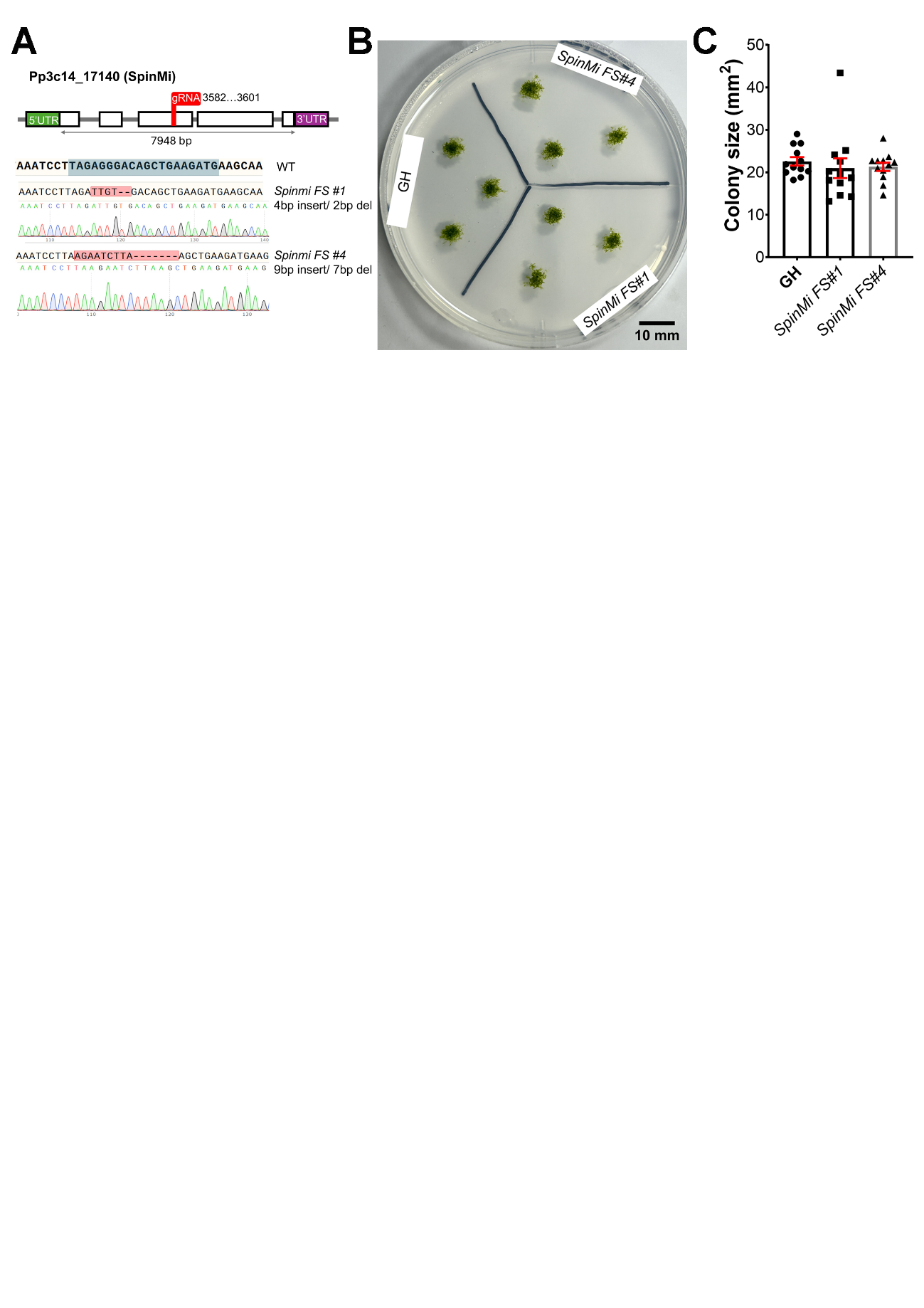


**Supplemental Figure 5. Frameshift mutations in *SpinMi* do not cause serious growth defects.** (**A**) Gene model of SpinMi and confirmation of frameshift mutations in the SpinMi gene by sequencing. The gRNA target site is indicated with a blue box. (**B**) Representative images of GH, *SpinMi FS#1* and *SpinMi FS#2* colonies after one month culture. (**C**) Colony size after one month of culture. No statistically significant difference found with one way ANOVA with Dunnett’s multiple comparison test against GH. Each data point corresponds to a single colony (mean±SEM).

**Supplemental Video 1. Cytokinesis failure was observed in *cyr1cyr2 #1* and *#2* CRISPR lines.** Live-cell imaging was performed in *P. patens* apical caulonemal cells in *cyr1cyr2 #1* and *cyr1cyr2 #2* CRISPR frameshift line expressing GFP-tubulin (green) and H2B-RFP (magenta). Images were acquired every 2 min in a single focal plane. Scale, 10 µm.

**Supplemental Video 2. Multiple phragmoplast formation *cyr1cyr2 #2* CRISPR line and CYR1-Citrine overexpression line.** Live-cell imaging was performed in *P. patens* apical caulonemal cells in *cyr1cyr2 #2* CRISPR frameshift line, expressing GFP-tubulin (left panel, green) and H2B-RFP (left panel, magenta), and CYR1-Citrine (right panel, green) overexpression line, which also expressed mCherry-tubulin (magenta, right panel) as a microtubule marker. Secondary phragmoplast formation is indicated with white arrows. Images were acquired every 2 min as a Z-stack (5 µm in 2.5 µm steps), best focal plane is shown. Scale, 10 µm.

**Supplemental Video 3. Localization of CYR1-Citrine and CYR2-Citrine.** Live-cell imaging was performed in *P. patens* apical caulonemal cells expressing mCherry-tubulin (magenta) and one of the following tagged proteins (green): CYR1-Citrine, CYR2-Citrine or Citrine. Images were acquired every 2 min as a Z-stack (5 µm in 2.5 µm steps), best focal plane is shown. Scale, 10 µm.

**Supplemental Video 4. Chromosome missegregation after inducible RNAi knockdown of LACH.** Representative images of mitotic progression and chromosome missegregation caused by depletion of LACH. RNAi was induced by addition of β-estradiol to the growth medium at final concentration of 1 µM, 5–6 days prior to observation. Images were acquired every 2 min as a Z-stack (5 µm in 2.5 µm steps); best focal plane is shown. Scale, 10 µm.

**Supplemental Video 5. Localization of SpinMi-mNeonGreen.** Live-cell imaging was performed in *P. patens* apical caulonemal cells expressing mCherry-tubulin (magenta) and SpinMi-mNeonGreen (green). Images were acquired every 2 min as a Z-stack (5 µm in 2.5 µm steps), best focal plane is shown. Scale, 10 µm.

**Supplemental Table 1.** Genes and gRNA library used for CRISPR genetic screening.

**Supplemental Table 2.** Results of *in silico* prediction of subcellular localization

**Supplemental Table 3.** *P. patens* transgenic lines, primers, and vectors used in this study
